# Clinical, Radiological, and Histological Assessment of Magnetic Nanoparticles as Pulpotomy Medicament in Primary Molars

**DOI:** 10.5005/jp-journals-10005-1527

**Published:** 2018-08-01

**Authors:** Harivinder R Konyala, Ajay R Mareddy, Niharika Puppala, N Venugopal Reddy, Manoj K Mallela, Keerthi P Susheela

**Affiliations:** 1 Reader, Department of Pedodontics, Mamata Dental College Khammam, Telangana, India; 2 Reader, Department of Pedodontics, Mamata Dental College Khammam, Telangana, India; 3 Senior Lecturer,Department of Pedodontics, Mamata Dental College Khammam, Telangana, India; 4 Professor, Department of Pedodontics, Mamata Dental College Khammam, Telangana, India; 5 Professor, Department of Pedodontics, SVS Institute of Dental Sciences Mahabubnagar, Telangana, India; 6 Senior Lecturer,Department of Pedodontics, Malla Reddy Institute of Dental Sciences, Hyderabad, Telangana, India

**Keywords:** Magnetic nanoparticles, Pulpotomy, Scanning electron microscope.

## Abstract

**Aim and objectives:**

Aim of the study was to evaluate the success of magnetic nanoparticles (MNPs) as pulpotomy medicament by clinical, radiologic, and histological assessment in primary molars.

**Materials and methods:**

The sample included 30 primary molars from 25 children aged between 3 and 9 years requiring pulpotomy treatment. Pulpotomy was carried out with MNPs. The teeth were evaluated after a period of 3, 6, and 12 months clinically and with the aid of radiographs. After 1 year, 10 teeth were extracted for histological evaluation under field emission scanning electron microscope (FE-SEM; ZEISS, Model No. Supra 55vp).

**Results:**

Of the samples, 98% showed clinical success rate with absence of pain, swelling, mobility, and abscess formation. After 3 months, 98% radiological success with absence of periodontal ligament widening, root resorption, and inter-radicular radiolucency was noted. Histological examination carried out under FE-SEM revealed a zone of odontoblastic proliferation at the interface between MNPs and odontoblastic layer of pulp and viable pulpal cells from the canal orifice till apical foramen.

**Conclusion:**

Magnetic nanoparticles can be recommended as an effective pulpotomy medicament with hard tissue barrier formation and preservation of vitality of remaining radicular pulp.

**How to cite this article:** Konyala HR, Mareddy AR, Puppala N, Reddy NV, Mallela MK, Susheela KP. Clinical, Radiological, and Histological Assessment of Magnetic Nanoparticles as Pulpotomy Medicament in Primary Molars. Int J Clin Pediatr Dent 2018;11(4):283-287.

## INTRODUCTION

Properties of magnetism are being used since decades in many medical applications. New advancements are being developed because of the availability of superior electromagnets, superconducting magnets, and permanent magnets.^1^

Nanostructured materials have unique electrical, chemical, structural, and magnetic properties, allowing them for use in information storage, biosensing applications, and biomedical engineering.^2^ Nanoparticles having magnetic properties carry great advantages by providing selective attachment to a functional molecule, confer magnetic properties to the target, and allow manipulation and transportation to a desired location through the control of a magnetic field produced by an electromagnet or permanent magnet. Magnetic nanoparticles consisting of cobalt, nickel, and neodymium-iron-boron offers improved magnetic properties.^3^

Medical applications include magnetic cell separation, immunoassay, hyperthermia, magnetic resonance imaging (MRI) contrast agents, target drug delivery, gene delivery, pathogen detection, and bone tissue engineering. One application is the use of nanomagnetic actuation for the mechanical conditioning of cells in tissue engineering and regenerative medicine.^4^ Several studies have reported their successful application in orthopaedics.^5,6^ The principle of pediatric endodontics in primary dentition is that tooth should remain in mouth in a nonpathological healthy condition to fulfill its role in primary and mixed dentition.^7^ Pulpotomy is the most common pulp treatment of primary molars.

Formocresol pulpotomy has been in use successfully since many years, but concerns over its toxicity and muta-genicity have impelled research into other pulpotomy techniques. Till now, many materials have been tried. Having known the scientific facts and the successful application of MNPs in orthopedics, this article presents a study where this novel material can be tried out as a medicament in pulpotomy of primary dentition.

## MATERIALS AND METHODS

This study was performed on 30 carious primary molars on 25 children aged between 3 and 9 years, who visited the Department of Pediatric Dentistry, with good general health and no history of systemic illness or hospitaliza-tion. Prior approval was obtained from the Institutional Ethical Committee before the commencement of the study.

The parents of the children signed informed consent after receiving detailed information regarding the procedure, benefits, and possible risks involved in the study. The pulpotomies were performed by one pediatric dentist under local anesthesia. The inclusion criteria,^8^ based on clinical and radiographic screening, were (1) teeth with deep carious lesion restorable after completion of the procedure (radiographically, the caries should be approximating to the pulp), (2) absence of symptoms indicative of advanced pulpal inflammation, such as spontaneous pain or history of nocturnal pain, (3) absence of clinical signs or symptoms suggesting a nonvital tooth, such as suppurating sinus soft tissue swelling, (4) absence of clinical radiographic signs of pulpal necrosis, i.e., furcation involvement, periapical pathology, internal resorption, calcification in canal, and (5) hemorrhage should stop within 5 minutes from the amputated pulp stumps using a sterile pledget of moist cotton.

After performing local anesthesia and isolation with rubber dam, soft debris, caries, and unsupported enamel and dentin were removed with large slow-speed round bur before pulpal exposure. Then, entire roof of the pulp chamber was removed. This procedure was accomplished using a No. 330 bur mounted in a water-cooled highspeed turbine. The coronal pulp was amputated to the canal orifices with a spoon excavator.

Pulp chamber was then irrigated with a light flow of normal saline. Hemostasis was obtained using sterile saline blotted sterile pellets. The pulp stump was covered with a paste of MNPs (Fe_3_O_4_ nanoparticles, 98%, alloy particle size (APS) < 40 nm, J.K Impex, Mumbai) prepared by mixing MNP powder with sterile saline using a 3:1 powder/saline ratio.

Over this, a thin dry collagen membrane (Collogen-esis, Salem) was placed to act as a barrier from the coronal restoration. The remaining pulp chamber was filled with a thick mix of zinc oxide eugenol (DPI, Mumbai, India). All teeth except those which were to be histologically evaluated were restored with a stainless steel crown (3M) cemented with glass ionomer cement (GC Fuji I, GC America, Alsip, Illinois, USA).

All teeth were clinically and radiographically evaluated at 3, 6, and 12 months interval by two unblinded standardized pediatric dentists. Restoration of pulpoto-mized deciduous molar teeth evaluation was carried out at baseline and control sessions under standard clinical conditions with a dental operating light, mouth mirror, and a dental explorer. They were examined regarding marginal discoloration, anatomic form, marginal integrity, and recurrent caries.

Clinically unacceptable situations were recorded and teeth with failed restoration were excluded from the study to ensure that the pulpotomy success rate was not affected. Teeth were considered to be clinically successful in the absence of spontaneous pain, draining fistula, swelling or abscess, mobility, premature exfoliation. Teeth were considered to be radiographically successful in the absence of abnormal root resorption, internal root resorption, pathological interradicular radiolucency, and calcification of canal. In this study, all children attended for follow-up evaluation.

Out of them, one-third of the sample (10 teeth) were further progressed for histological assessment under FE-SEM. Histological assessment was done on teeth, which were undergoing serial extraction procedure. After 3 months following pulpotomy procedure, the teeth were extracted and subjected for histological evaluation. Freshly extracted carious teeth were considered as controls.

All the extracted teeth were fixed in buffered formalin 10% for 48 hours. Then, each specimen was washed in sodium phosphate buffer of concentrated 0.05 M at pH -7.4, dehydrated under series of alcohol (10-100% for 10 minutes each), followed by air drying. Samples were then fixed to stub with a carbon tape and mounted for gold sputtering.

They were then coated with gold alloy (40% gold) which was ready for examination under FE-SEM. The sections were blindly evaluated by experienced pathologists and were calibrated according to the criteria^9^ described as follows: Inflammatory cell response was as follows. Grade I: absent or very few inflammatory cells. Grade II: mild or average number less than 10 inflammatory cells. Grade III: severe inflammatory lesion appearing as an abscess or dense infiltrate involving one-third or more of the coronal pulp. Grade IV: completely necrotic pulp. Dentin bridge formation: grade I: presence of a dentin bridge directly adjacent to some portion of the medicament interface. Grade II: presence of a dentin bridge distant from the medicament interface grade III: no evidence of any dentin bridge formation in any sections.

The scores attributed were subjected to chi-square test. A “p” value of <0.05 was considered as statistically significant.

## RESULTS

Thirty teeth (18 girls, 12 boys) were included in the present study and were treated at the beginning of the study. Two teeth were lost to follow-up at the end of 3 months. Thus, 28 teeth were evaluated clinically and radiographically at the end of 3 months.

**Figs 1A to C:**
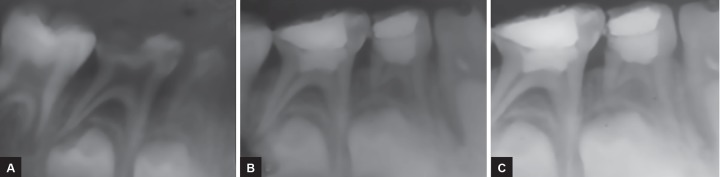
(A) Preoperative radiograph, (B) postoperative radiograph, and (C) 1-year follow-up radiograph

Clinical evaluation did not show any signs and symptoms of inflammation in any case at the end of 3 months, except for one case which presented with buccal abscess. In radiographic evaluation, no signs of failure were noted at 2 months interval, except for one case which showed radiolucency in furcation area. Similar results were obtained at the end of 6 and 12 months (Fig. 1). The overall clinical and radiographic success rate was 98%.

**Figs 2A to D:**
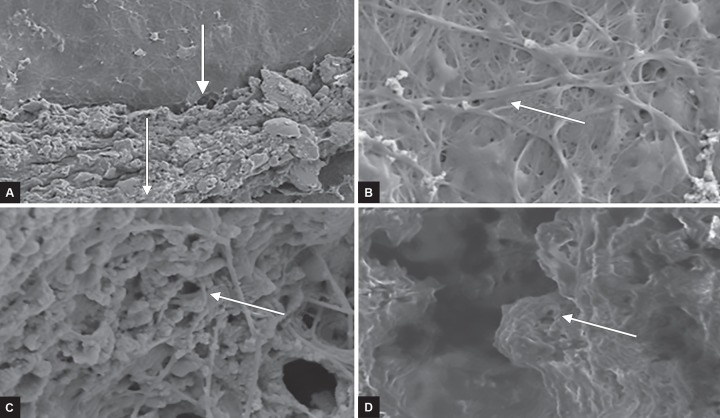
Photomicrographs of samples under FE-SEM. (A) Photomicrograph showing cross-section of newly formed dentin bridge adjacent to MNP layer. (B) Photomicrograph showing proliferation of osteoblasts with good interlacing of viable connective tissue with normal cell distribution, morphology, and cytoskeleton organization. (C) Magnified view of (B). (D) Magnified view of odontoblastic layer

Histological evaluation: Histological examination of all the samples under FE-SEM revealed a zone of odontoblas-tic proliferation at the interface between MNPs and odon-toblastic layer of pulp and presence of viable pulpal cells with interlacing collagen fibers from the canal orifice to apical foramen (Fig. 2). This could be considered as dentin bridge representing the pulpotomized regenerating site.

## DISCUSSION

The MNPs are biocompatible^10^ and have been tried in many medical applications and were proved to be successful, and their biomedical applications include MRI, hypothermic treatment for malignant cells, target drug delivery, bio-detection of pathogens and most importantly for bone tissue engineering.^1^ The MNPs have been tried for osteogenesis in a study which showed good cell adhesion and proliferation under SEM.^11^ They positively influence osteoblast-like cell behavior, suggesting them to be promising biomaterial for regeneration.^12^ These MNPs also possess an inhibitory effect on the growth rate of *Streptococcus mitis, Streptococcus salivarius, Staphylococcus aureus, Candida albicans,* and *Enterococci* when static magnetic field of 50 to 150 G is applied on oral mucosa.^13^ Its mechanism of action could be explained by Wang-Ingber’s concept which states that MNPs apply mechanical forces directly to cells by attaching to cell membrane, and when presented on cell surface, they are engulfed with the action of integrin receptors (extracellular protrusion of cytoskeleton) on cell surface; when a strong magnetic field is created, these MNPs experience translational forces on receptors which pull the particles to a targeted site, activating mechanosensitive ion channels. This mechanism has an advantage that one ion channel can be activated without interfering in the normal functioning of other ion channels in cell membrane. Such a binding provides potential for independent activation of cellular functions with a high degree of specificity.^3^

In the present study, –320 mT MNPs were used which showed excellent proliferation of osteoblasts with good interlacing of viable connective tissue. The cell distribution, morphology, and cytoskeleton organization were unaltered with the typical distribution of cytoplasmic actin fibrils similar to the findings of Cunha et al.^14^ The SEM observations showed a positive correlation of MNPs influencing dental pulp stem cells (DPSCs) in the formation of intertubular dentin in all the samples which were in accordance with the observations of Juliana et al^15^ who demonstrated that the DPSCs differentiated into osteoblasts, odontoblast, and resulted in dentinal repair.

The expression of dentin sialophosphoprotein and osteocalcin proves that both odontoblast and osteoblast cells exist in human pulp tissues.^15^ Therefore, this material possesses a regenerative capacity which is a novel approach unlike the devitalization technique (formocre-sol pulpotomy) used conventionally which is proven to be toxic by many studies.^16,17^ The benefits of this novel material are a gift to the human body.

The particle size of MNP enhances the material to have the ability to enclose proximity to a specific biological entity and interact at cellular subcellular protein/genetic scale. As MNPs have multifunctional aspect, they can be used in tagging, tracking, and activation of cells both *in vivo* and *in vitro* and attract particles to the chosen site and hold them there until therapy is complete.

When entering into blood circulation, they are physiologically well tolerated and have no measurable toxicity; they do not affect the viability of cells unlike the conventional materials used for pulpotomy.^18^ Differentiation of cells is an exciting potential for MNPs technology and field of tissue engineering, as they are useful for stimulating specific cells, which was observed in our study.

## CONCLUSION

Therefore, from the findings of our study, it can be concluded that MNPs can be an ideal and novel pulpotomy medicament with hard tissue barrier formation and preservation of vitality of remaining radicular pulp, but further studies are necessary with a larger sample size and long-term follow-up to evaluate the success rate and for the material to be commercially used on a large scale.
